# The effect of high dietary fiber intake on gestational weight gain, fat accrual, and postpartum weight retention: a randomized clinical trial

**DOI:** 10.1186/s12884-020-03016-5

**Published:** 2020-05-24

**Authors:** Holly R. Hull, Amy Herman, Heather Gibbs, Byron Gajewski, Kelli Krase, Susan E. Carlson, Debra K. Sullivan, Jeannine Goetz

**Affiliations:** 1 Department of Dietetics and Nutrition, University of Kansas Medical Center, 3901 Rainbow BLVD, MS 4013, Kansas City, KS 66160 USA; 2 Department of Biostatistics & Data Science, University of Kansas Medical Center, Kansas City, KS USA; 3 Department of Obstetrics and Gynecology, University of Kansas Hospital, Kansas City, KS USA

**Keywords:** Excessive weight gain, Single goal intervention, High fiber, Pregnancy, Body fat, Dietary fiber

## Abstract

**Background:**

Interventions to prevent excessive gestational weight gain (GWG) have had limited success This pilot study examined the effectiveness of a single goal (SG) high dietary fiber intervention to prevent excessive GWG.

**Methods:**

Twelve weekly lessons focused on consuming a high fiber diet (≥30 g/day). Snacks containing 10–12 g of dietary fiber were given for the first 6 weeks only. Body composition was measured at baseline and at the end of the intervention. At one-year postpartum, body weight retention and dietary practices were assessed. A *p*-value is reported for the primary analysis only. For all other comparisons, Cohen’s d is reported to indicate effect size.

**Results:**

The SG group increased fiber intake during the study (32 g/day at 6 weeks, 27 g/day at 12 weeks), whereas the UC group did not (~ 17 g/day). No differences were found for the proportion of women classified as excessive gainers (*p* = 0.13). During the intervention, the SG group gained less body weight (− 4.1 kg) and less fat mass (− 2.8 kg) (d = 1.3). At 1 year postpartum, the SG group retained less weight (0.35 vs. 4.4 kg, respectively, d = 1.8), and reported trying to currently eat high fiber foods.

**Conclusion:**

The SG intervention resulted in less weight gain, fat accrual, and weight retention at 1 year postpartum. A residual intervention effect was detected postpartum with the participants reporting continued efforts to consume a high fiber diet.

**Trial registration:**

NCT03984630; Trial registered June 13, 2019 (retrospectively registered).

## Background

Overall, 55% of women gain excessive gestational weight [1] and diet quality decreases across pregnancy and into the postpartum period [2]. Maternal excessive gestational weight gain (GWG) and low diet quality are associated with poor offspring [3] and maternal outcomes [4]. At 1 year post-pregnancy, excessive GWG shifts 33% of normal weight women into an overweight or obese BMI category and 44% of overweight women into an obese category [5]. Excessive GWG, experienced by 56% of U.S. pregnant women [1, 6], increases offspring fat accrual and risk for overweight or obesity development, a characteristic of 31.8% of US children [7]. Excess adiposity drives disease development [8], leading to the prediction of a rapid generational decline in life expectancy [9] and a surge in medical care costs (~$150 billion/yr) [10].

Pregnancy is a time when pregnant women are open to adopt healthy behaviors for the well-being of their baby [11]. However, the best intervention approach to achieve this goal is not clear. Limited success has been reported in published interventions [12, 13]. Failed studies cite poor adherence as a contributor to a lack of intervention success [14, 15]^.^

Adherence is one of the strongest predictors of success in weight management studies [16]. Complex dietary interventions requiring a high level of literacy to follow (e.g. low glycemic index diet) [17] or interventions requiring change in multiple behaviors have poor adherence rates [12]. In non-pregnant populations, interventions based on diet only have better adherence and long-term behavior adoption [12, 18]. The better effectiveness is hypothesized to be because the multiple goal approach requires focus on several messages resulting in the intervention intensity being diluted [12]. Further, better outcomes in the single goal (SG) interventions may be related to specific dietary components protecting against weight gain that have not yet been explored [12].

One nutrient that exerts a beneficial effect on controlling body weight is dietary fiber. Dietary fiber aids in weight loss and maintenance [19], promotes satiety and reduces hunger [20], reduces inflammation [21], and exerts clinical benefits by controlling glucose, insulin levels and lipid levels [22]. Fiber is a powerful prebiotic that changes the gut microbiota [23], gastrointestinal processes (e.g., gastric emptying rate, small intestine and colonic transit time, and intestinal permeability) [24], and the microbial metabolome [25] that all favorably impact GWG, metabolism, inflammation, and appetite. Gut microbiota dysbiosis is found in pregnant women that develop pregnancy complications [26, 27] and is related to GWG^28^ and metabolic biomarkers and inflammation [28]. The maternal microbiome is identified as a therapeutic target to improve the health of pregnant women and their offspring that is widely understudied [28, 29]. The current dietary fiber intake in the US during pregnancy is low, 17.3 g/day [30], which is well below the recommended intake of 28 g/day [31]. Therefore, increasing fiber intake during pregnancy has the potential to have a large and beneficial impact.

We designed a single goal focused study to improve adherence and long-term behavior adoption that had a high likelihood for a significant physiologic and metabolic impact. Therefore, the primary aim was to compare the proportion of women gaining excessively between a SG high fiber diet (> 30 g/day) versus usual care (UC). Secondary outcomes compared between group differences for total GWG and fiber intake. In addition, we compared between groups differences for maternal fat accretion during pregnancy, post-partum weight retention, and explored if an intervention effect could be detected at 1 year postpartum.

## Methods

### Design overview

This pilot study was a randomized clinical trial to assess the effectiveness of a SG high dietary fiber (≥30 g/day) intervention to prevent excessive GWG compared to the UC group. The intervention consisted of 12 weekly 60-min lessons led by a Registered Dietitian aimed to increase fiber intake and was delivered using group-based phone counseling. The study was approved by the University of Kansas Medical Center Institutional Review Board (#00004032) and registered at ClinicalTrials.gov (NCT03984630). The trial was registered retrospectively. All subjects provided written informed consent prior to study participation.

### Subjects and randomization

**Fig. 1:**
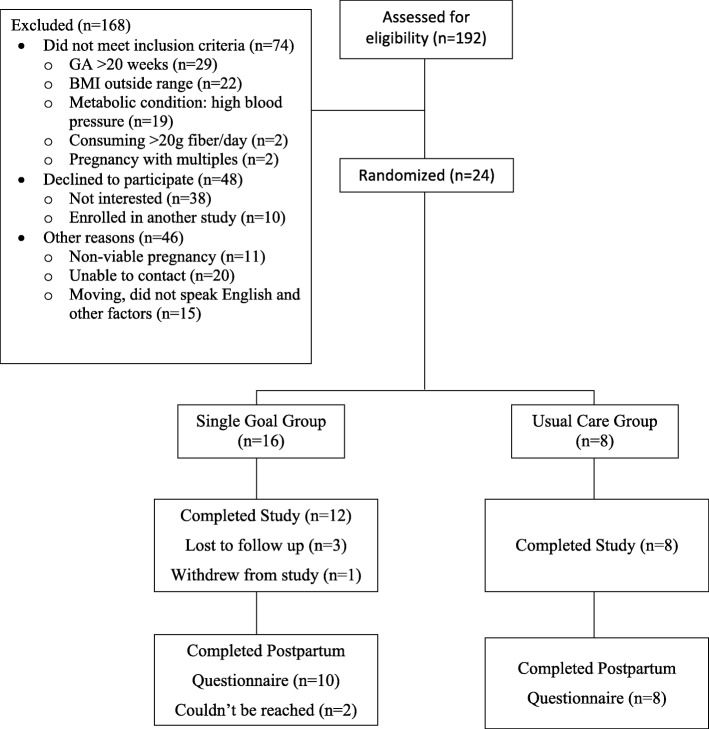
Consort Diagram

Women were recruited between 10 and 14 weeks in gestation in three waves between August 2016 and December 2016. Participants were block randomized in groups of 6–10 into either the intervention or the UC group at a 2:1 ratio. Randomization was computer-generated using excel software by the study statistician. Participant blinding was not possible, but study staff taking assessments were blinded to group assignment. The inclusion criteria were maternal age 18–45 years, singleton pregnancy, and body mass index (BMI) ≥22 kg/m^2^–40 kg/m^2^. We focused on women with a BMI ≥22 kg/m^2^ because they have a greater likelihood of gaining excessively, retaining weight postpartum, and shifting their BMI to an overweight or obese BMI category post-pregnancy [1, 32]. Women were excluded if they had pre-gestational diabetes, gestational diabetes, pre-eclampsia, hypertension, other metabolic abnormalities, heart disease, smoking, and drug abuse. No women developed any of these medical conditions during the 12-week intervention. A CONSORT diagram is included in Fig. 1.

### Single goal intervention

The intervention group was instructed to consume ≥30 g fiber/day but was not given a calorie goal. This was intentional because we wanted to remove the driver of what is known to induce weight loss (calorie goal) and determine if a fiber goal alone can prevent excess GWG. The curriculum was developed based on the theoretical framework of the social cognitive theory (SCT) [33] and focused on behavior shaping, goal setting, feedback and reinforcement, social/peer support, stimulus control, and relapse prevention. Participants received a binder with all lesson materials and were taught to track their daily total fiber intake using the *LSTAtHome* App (LifeScience Technologies, LLC, www.lifesciencetechnologies.com). In the App, only feedback on fiber intake was provided. No information was visible to the participants for kcals, other macronutrients than fiber, or micronutrients. The curriculum encouraged a balanced diet emphasizing fruits and vegetables, whole grains, low-fat dairy, and lean protein. Lessons focused on how to increase fiber intake with education on foods that contain fiber, high fiber recipes, and how to make over recipes to increase dietary fiber content. Sample weekly menu plans with grocery and shopping guides were provided. General physical activity recommendations during pregnancy were mentioned but no focus or reinforcement was provided (no pedometers).

Participants were given a phone number with a unique access code that allowed them to enter the group session. Maestro (Oakland, CA; www. maestroconference.com) phone conference system was used and each call was recorded. The session started with a review of the prior week’s goals, whether the goal was met, and a self-reflection of why the goal was or was not met with support and encouragement from group members and the Dietitian. Each week there was a structured lesson with an assignment to be completed after the session. The session ended with goal setting, discussion, questions, and wrap-up.

### Snacks high in dietary fiber

To aid in increasing dietary fiber intake while skills to increase daily fiber intake were learned in weekly lessons, intervention participants received 6 weeks of high fiber snacks to consume two times per day. Each snack had ≥3 g fiber/100 kcal. The daily fiber total for the two snacks was 10–12 g of fiber ranging in kcal from 210 to 380 kcals. The snacks consisted of shelf stable foods that were given to the participant at the baseline visit. Examples include multiple flavors of Kind bars, chickpeas, chips made from beans and lentils, and snap peas. Additionally, they received whole grain cereals including whole wheat Puffins, Krave, and Chex.

### Weekly body weight

Both groups were given body weight scales and standardized instructions with details on measuring body weight. Participants were instructed to measure body weight on the same day of the week at the same time of day while wearing minimal clothing and after voiding. Body weight was reported weekly either through the *LST at home* App for the intervention group or by text or email for the UC group.

### Dietary recall

Three multiple-pass 24-h dietary recalls (two weekdays and one weekend day) were collected at baseline, 6 weeks, and 12 weeks, by trained research staff to characterize energy and nutrient intake. Multiple-pass 24-h recalls accurately estimate dietary intake [34, 35] and contain less reporting bias than diet records [34, 36]. The recalls were entered into the Nutrition Data System for Research (NDS-R, version 2016, Minneapolis, MN) for macro- and micronutrient analysis. For the baseline and 12 week visit, one diet recall occurred in person at the study visit. For the 6 week recalls, all occurred via the phone.

### Height and weight

Height was measured without shoes to the nearest 0.1 cm using a wall mounted stadiometer (Health o meter®, Bradford, MA) at the baseline visit using standardized procedures. Body weight was measured while the participant was wearing minimal clothing (Seca 869, Chino, CA). Pre-pregnancy BMI was calculated using the measured height and the self-reported pre-pregnancy body weight.

### Gestational weight gain calculation

Body weight was assessed in the laboratory at the baseline visit (week 0) and again after the intervention. These two values were used to calculate weight gain during the intervention. At study enrollment, women self-reported their pre-pregnancy body weight. Women were contacted following delivery and reported the highest body weight measured during their pregnancy. These values were used to calculate total GWG. The 2009 IOM GWG guidelines [37] classifies excessive GWG as the following for each pre-pregnancy BMI group: normal weight women > 16 kg, overweight women 11.5 kg, and obese women > 9 kg. In addition, the IOM GWG guidelines lists expected GWG during each trimester. A personalized weight gain range was calculated based on the gestational week the participant started and ended the intervention. If the GWG during the intervention was above the calculated range, her weight gain was categorized as excessive using published ranges [37].

### Body composition

Maternal body composition was assessed at baseline and at the completion of the study. The three-component model of Siri et al. [38] was used to estimate body composition. The Siri et al. model uses total body water (TBW) measured by bioelectrical impedance, body volume (BV) measured by the Bod Pod® and body weight. Fat mass (kg) was determined by the following equation: FM (kg) = 2.057*BV (L) - 0.786*W (L) – 1.286*BW (kg); where BV is body volume in liters, W is TBW in liters, and BW is body weight in kilograms. Percentage of body fat (% fat) was calculated as (FM/BW)*100%. Body composition testing was completed following a standardized testing protocol uniform to our Body Composition Laboratory for all populations. Briefly, women reported to the laboratory for body composition assessment after a 4 hour fast and refraining from exercise for 12 h. All tests were completed on the same day. Body volume was assessed using air displacement plethysmography (Bod Pod; COSMED; Concord, CA) with subjects wearing a tight-fitting one-piece swimsuit and a fitted hat (Allentown Scientific Associates, Inc., Allentown, PA). Body weight was assessed on body weight scale (Seca, Inc., Chino, CA) and this value was used in the Siri equation to estimate body fat. Total body water was assessed by bioelectrical impedance (Tanita, Inc., TBF-310, Tokyo, Japan).

### Postpartum questionnaire

Women were emailed a questionnaire via RedCap [39] to report their body weight at 1 year postpartum. Postpartum weight retention was calculated by subtracting the pre-pregnancy body weight obtained during the study from the reported body weight at 1 year postpartum. In addition, questions were asked to assess if there were any residual behaviors from the intervention maintained postpartum and what information learned during intervention was still being used. Women were asked the following question: “*Do you currently use information from the intervention to guide your eating*” (response yes or no), “*What information do you use from the intervention to guide your eating*?” (open ended response), and “*Do you currently try to eat high fiber foods as part of your diet*?” (response yes or no).

### Statistical analysis

The power calculation was based on a prior multiple goal (MG) pilot study performed by the study team where 78% gained excessively in the control and 29% gained excessively in the MG arm (unpublished data). Using this effect size, a sample size of *n* = 13 women per arm (1:1) was estimated to have 83% statistical power with significance set a one-sided Type I error < 0.05 using a Chi-squared test to detect a difference between the proportion of women gaining excessively in the SG intervention and the control. Though we lost no subjects in the prior pilot, we factored in 20% attrition, resulting in *n* = 16 women being recruited for the SG arm. A 2 to 1 unequal allocation of women was employed, resulting in *n* = 16 randomized to the SG arm and *n* = 8 randomized to the control (UC) arm. Four (all in the SG group) were lost to follow-up, therefore, the final administered power for the primary aim only, was calculated to be 72%. No differences were found between the four lost to follow-up and those that completed the study.

Means and standard deviations were calculated for all continuous variables. To determine if the proportion of women gaining excessively differed between groups, a Chi-square test was completed at the end of the intervention (12 weeks) and at the end of pregnancy. An ANCOVA assessed if there was a between group difference for the change in body weight (during the intervention, total GWG, and up to 1 year postpartum) and body composition. The confounding variables included in the model were time between the baseline and 12 week visits, 6 week and 12 week total dietary fiber intake (grams/day), maternal age, and parity. A one-way ANOVA assessed between group (UC vs SG) differences at each study time point: week zero (baseline), week 6, and week 12. A paired t-test assessed within group differences between each study time point. Analyses are presented for all completers (intent to treat) and by using compliant subjects. Compliance was determined by attendance at the weekly GBPC sessions. To be considered compliant, participants must have attended ≥65% of the sessions (8 of 12 lessons). SPSS (IBM, version 24) was used for data analysis and significance was set at *p* ≤ 0.05 for the primary analysis only. For all other comparisons, we calculated Cohen’s d to indicate effect size [40]. For this report, the values indicate how large of an effect was found in the intervention relative to the control group. Cohen’s d values are: ≤0.2 = negligible effect, > 0.2 to ≤0.5 = small effect, > 0.5 to ≤0.8 = medium effect and > 0.8 = large effect.

## Results

Twenty-four women were enrolled and randomized to the SG (*n* = 16) or UC (*n* = 8). In the SG group, three women were lost to follow up and one woman withdrew, therefore, a total of *n* = 12 women completed the SG intervention. All women from the UC group completed the study. The average participant age was 29.4 ± 3.7 years and the group had a mean pre-pregnancy BMI of 27.0 kg/m^2^ (SD 5.3). Baseline characteristics are presented in Table 1. A greater proportion of women in the UC group had one or more prior pregnancies (40%) when compared to the SG group (17%; d = 0.7). No unintended side effects or adverse events were reported.

**Table 1:** Maternal descriptive characteristics at baseline

Characteristic	Control (***n*** = 8)	Intervention (***n*** = 12)	Cohen’s d
Age (years)	30.5 ± 3.2	29.0 ± 3.5	0.4
Pre-pregnancy BMI (kg/m^2^)	26.8 ± 5.7	26.3 ± 5.7	0.1
Height (cm)	163.2 ± 6.5	161.2 ± 6.6	0.3
Body weight (kg)	73.7 ± 13.5	70.7 ± 18.5	0.2
Percentage body fat (%fat)	30.9 ± 5.9	28.9 ± 7.8	0.3
Fat mass (kg)	23.4 ± 8.3	21.6 ± 12.3	0.2
Fat-free mass (kg)	50.1 ± 5.0	48.9 ± 6.4	0.2
Dietary fiber intake (g/day)	18.3 ± 7.7	21.2 ± 7.4	0.4
Energy intake (kcal/day)	1714.8 ± 692.8	2038.8 ± 361.9	0.6
Energy from carbohydrate (%)	50.8 ± 8.8	46.5 ± 7.8	0.5
Energy from fat (%)	32.3 ± 5.3	37.9 ± 7.5	0.9
Energy from protein (%)	16.9 ± 4.6	15.4 ± 2.5	0.4
Parity			
First pregnancy	3	10	
One or more prior pregnancies	5	2	1.2
Ethnicity			
Non-Hispanic or Latino	7	12	
Hispanic or Latino	1	0	N/A
Education			
High school or less	1	1	
Some college or graduated college	1	7	
Graduate degree	6	4	1.0
Household income (dollars)			
< $50,000	1	1	
$50,000 - $74,999	1	4	
$75,000 - $99,999	1	2	
$100,000 - $124,999	4	4	0.4
> $125,000	1	1	

Legend: Mean ± standard deviation

### Adherence to GWG recommendations, GWG, and body composition changes

Adherence to the 2009 GWG guidelines was calculated as excessive or not excessive for during the intervention and for total GWG (Table 2). For the primary analysis, though fewer women in the SG intervention gained excessively, no between group differences were found for the proportion of women classified as excessive gainers during the study (62% vs. 42%; *p* = 0.36) or at end of pregnancy for total GWG (75% vs. 42%; *p* = 0.13). However, large effect sizes were found for between group differences for weight gain and fat accrual. Table 3 presents the changes in body weight during the intervention, total GWG, and changes in body composition. During the intervention, the SG group gained less body weight (− 3.8 kg; d = 1.2) and less fat mass (− 2.8 kg; d = 1.3). Large differences were found in body weight gain and fat mass accrual during the intervention among the completers and the compliance analysis. In the completers (*n* = 20), the SG group had 8.4 kg less total GWG (20.5 kg vs. 12.1 kg; d = 1.3) while the difference was 5.4 kg less total GWG using subjects who were classified as compliant (*n* = 16; 18.5 kg vs. 13.1 kg; d = 0.8).

**Table 2:** Percentage of excessive GWG at the end of the intervention (12 weeks) and at the end of pregnancy

Variable	*All completers*			*Compliance analysis*		
	Usual care (***n*** = 8)	Single goal (***n*** = 12)	Cohen’s d	Usual care (***n*** = 8)	Single goal (***n*** = 8)	Cohen’s d
Percentage excessive at 12 wks, n (%)	5 (63%)	5 (42%)	d = 0.5	5 (62%)	2 (25%)	d = 0.9
Percentage not excessive 12 wks, n (%)	3 (38%)	7 (58%)		3 (38%)	6 (75%)	
Percentage excessive at end of pregnancy, n (%)	6 (75%)	5 (42%)	d = 0.8	6 (75%)	3 (38%)	d = 0.9
Percentage not excessive end of pregnancy, n (%)	2 (25%)	7 (58%)		2 (25%)	5 (62%)

**Table 3:** Changes in body weight, body composition, and percentage of excessive GWG at the end of the intervention (12 weeks) and at the end of pregnancy

Variable	*All completers*			*Compliance analysis*		
	Usual care (***n*** = 8)	Single goal (***n*** = 12)	Cohen’s d	Usual care (***n*** = 8)	Single goal (***n*** = 8)	Cohen’s d
Body weight change baseline to 12 wks (kg)^a^	8.0 ± 3.4	4.2 ± 3.2	1.2	7.7 ± 3.2	3.0 ± 2.1	1.8
Total gestational weight gain (kg)^b^	20.5 ± 6.9	12.1 ± 6.5	1.3	18.5 ± 7.1	13.1 ± 6.9	0.8
Change in percentage fat mass baseline to 12 wks (%fat)^a^	3.9 ± 1.9	2.0 ± 1.7	1.1	3.9 ± 1.9	1.6 ± 1.3	1.4
Change in fat mass baseline to 12 wks (kg)^a^	5.4 ± 2.2	2.6 ± 2.1	1.3	5.2 ± 2.1	1.9 ± 1.1	2.1
Change in fat-free mass baseline to 12 wks (kg)^a^	2.6 ± 1.7	1.6 ± 1.6	0.6	2.5 ± 2.6	1.1 ± 2.5	0.5

Legend: Mean ± standard deviation;

^a^covariates included in the model: between testing visits, 6 wk. total fiber intake (g), and 12 wk. total fiber intake (g), parity, and maternal age;

^b^covariates included in the model: 6 wk. total fiber intake (g), and 12 wk. total fiber intake (g), parity, and maternal age

### Changes in dietary intake during the intervention

Dietary intake data and analyses were similar whether analyzing all completers or by those considered compliant. Dietary intake data are presented only for those classified as compliant, and receiving the a priori intervention dose (Table 4; *n* = 16). Calories/day or % kcal from carbohydrate, protein, or fat were similar between groups (d = 0.0–0.5). Large effects were found for total fiber intake/day, soluble fiber, insoluble fiber, and fiber grams/100 kcal/day. The SG group maintained an increased fiber intake throughout the study (27 to 32 g/day), whereas the fiber intake for the UC group was unchanged during the study (~ 17 g/day). An increased fiber intake was maintained without a significant increase in energy intake. This suggests the increase in fiber intake was achieved by consumption of nutrient dense foods, versus achieving a fiber intake with a higher energy intake.

**Table 4:** Within and between group differences for dietary intake

Study time point	Group		Between group effect sizes
	***UC (n = 8)***	***SG (n = 8)***	Cohen’s d
*Calories (kcal/day)*			
*Week 0*	1714.8 ± 692.8	1941.9 ± 406.9	0.4
*Week 6*	1710.7 ± 794.1	1984.1 ± 280.6	0.5
*Week 12*	1622.1 ± 623.1	1853.2 ± 307.9	0.5
*% kcal from carbohydrate*			
*Week 0*	50.8 ± 8.8	47.6 ± 8.3	0.4
*Week 6*	49.2 ± 10.7	49.5 ± 4.5	0.0
*Week 12*	51.8 ± 13.1	50.4 ± 7.0	0.1
*% kcal from protein*			
*Week 0*	16.9 ± 4.6	15.0 ± 2.2	0.6
*Week 6*	16.5 ± 4.7	15.2 ± 3.0	0.3
*Week 12*	17.0 ± 4.5	15.4 ± 3.2	0.4
*%kcal from fat*			
*Week 0*	32.3 ± 5.3	37.3 ± 9.0	0.7
*Week 6*	34.2 ± 7.6	35.3 ± 2.8	0.2
*Week 12*	31.2 ± 10.4	34.1 ± 4.3	0.4
*Fiber intake (g/day)*			
*Week 0*	18.3 ± 7.8	21.0 ± 7.0	0.4
*Week 6*	16.7 ± 8.9	32.1 ± 9.7	1.7
*Week 12*	16.7 ± 4.9	27.0 ± 8.9	1.5
*Fiber grams/100 kcal (g/100 kcal/day)*			
*Week 0*	1.10 ± 0.31	1.09 ± 0.29	0.0
*Week 6*	0.97 ± 0.36	1.60 ± 0.39	1.7
*Week 12*	1.10 ± 0.35	1.44 ± 0.33	1.0
*Soluble fiber intake (g/day)*			
*Week 0*	5.5 ± 2.3	6.6 ± 2.3	0.5
*Week 6*	4.8 ± 2.5	10.0 ± 2.6	2.2
*Week 12*	4.8 ± 1.9	7.8 ± 1.8	1.6
*Insoluble fiber intake (g/day)*			
*Week 0*	12.7 ± 6.1	14.4 ± 6.4	0.3
*Week 6*	11.8 ± 6.5	21.7 ± 8.0	1.4
*Week 12*	11.7 ± 3.8	18.8 ± 7.5	1.3

Legend: Within group differences (e.g., SG week 0 different from SG week 6):

### Postpartum questionnaire

The amount of weight retained at 1 year postpartum is presented in Table 5. Large effect sizes were found between groups for weight retained at 1 year postpartum (d= > 1.8) with the SG group retaining ~ 4 kg less body weight. For all completers in the SG group (*n* = 12), ten women completed the postpartum survey. Participants in the SG group were asked: “*Do you currently use information from the intervention to guide your eating*”. Thirty percent of SG participants reported using information from the intervention. Women were asked the open-ended question: “*What information do you use from the intervention to guide your eating*?” Responses included: high fiber snack ideas (*n* = 3), high fiber meal ideas (*n* = 3), continue to consume some of the snacks provided during the study (*n* = 2), how to check amount of fiber in foods (*n* = 1), and portion control (*n* = 1). Participants in the SG group were asked: “*Do you currently try to eat high fiber foods as part of your diet*?”. All ten women reported trying to currently eat high fiber foods as part of their diet.

**Table 5:** Weight retained at one year postpartum

Variable	*All completers*			*Compliance analysis*		
	Usual care (***n*** = 6)	Single goal (***n*** = 10)	Cohen’s d	Usual care (***n*** = 6)	Single goal (***n*** = 7)	Cohen’s d
Weight retained at one year postpartum (kg)	4.4 ± 2.6	0.35 ± 1.8	1.8	4.4 ± 1.9	−0.9 ± 1.8	2.9

Legend: Mean ± standard deviation; covariates included in the model: 6 wk. total fiber intake (g), 12 wk. total fiber intake (g), parity, and maternal age

## Discussion

Fewer women in the SG group had excessive GWG during pregnancy, however, no significant between group differences were found during the intervention or at the end of pregnancy. Nonetheless, SG participants gained less body weight, fat mass, and retained less body weight at 1 year postpartum. The magnitude of the between group difference for body weight gained (3.8 kg) and fat accrued (2.8 kg) was large both during the intervention, for total GWG (8.4 kg), and postpartum weight retention (> 4 kg). This is encouraging considering the intervention started at the end of the first trimester (~ 13 weeks in pregnancy) and concluded at end of the second trimester (~ 25 weeks). Therefore, women were without contact or follow up during the last trimester of pregnancy. Further, behaviors learned during the intervention were still being used by the women in the SG group. Ten of the 12 women completed the postpartum follow up survey. All ten women reported continued efforts to consume a high fiber diet. Given the small sample size and short duration of this pilot study, this difference is noteworthy, clinically meaningful, and a promising study design that appears to have a continued effect detected at 1 year postpartum.

Limited data are available regarding fiber intake and GWG during pregnancy and no RCTs have been reported. Pregnancy represents a transient excursion into a metabolic syndrome like state [41]. In non-pregnant adults with metabolic syndrome, a large NIH funded RCT study (R01 HL094575) [18] compared the effectiveness a multiple goal (gold standard for weight loss) vs. SG high fiber intervention for weight loss and metabolic changes. Both groups saw similar improvements in weight loss, dietary quality, insulin resistance, lipids, inflammation, glucose levels, and blood pressure and the interventions were concluded to be equivalent.

Though no studies have been reported using a high fiber diet during pregnancy, data are available from low glycemic index (GI) dietary studies. The GI characterizes the capability of carbohydrates to raise blood glucose levels and high fiber foods score low on the GI. Thus, a low GI diet should be higher in dietary fiber. The Randomized Control Trial of Low Glycemic Index Diet (ROLO) study compared a low GI diet to usual care for prevention of macrosomia and excessive maternal GWG [42]. No differences were found between groups for infant birth outcomes. However, the low GI group gained less weight (12.2 vs. 13.7 kg; *p* = 0.01), had lower rates of excessive GWG (38% vs. 48%; *p* = 0.01), and had greater fiber intake (20.3 vs. 18.8 g; *p* < 0.001) [43]. A small benefit was found on maternal insulin levels early in pregnancy only, however, no effect was found for leptin or markers of inflammation. Follow-up data from the ROLO trial during the first 6 months and five-years postpartum found a limited sustained intervention effect [44–46]. Greater weight loss was found at 3 months postpartum, however, this difference was not detected at 6 months or 5 years postpartum. At 3 months postpartum, a greater number of women from the intervention reported consulting food labels to read nutrient values, however, this was no longer detected at 6 months or 5 years postpartum.

In our study, we found the SG group gained less body weight, accrued less fat mass, and retained less body weight postpartum. Further, women who participated in the SG intervention reported continued efforts to eat a high fiber diet and using skills taught during the intervention at 1 year postpartum. One potential reason for the difference between studies may be the complexity and resources needed to follow a low GI diet. Following a low GI diet takes a high level of health literacy, access healthcare providers, nutrition information, training on the GI, and numeracy skills. The effect in our study during pregnancy and at 1 year postpartum may be greater because of the simplicity of a SG high fiber intervention to adhere to during pregnancy and follow long-term.

Adherence is the strongest predictor of success in adult weight management studies [16] and is likely an important predictor of success in interventions to prevent excessive GWG. The SG high fiber intervention may have increased short and long-term adherence over other dietary and intervention approaches due to the simplicity of the SG message. A SG intervention sets one goal with repeated reinforcement, compared to interventions where multiple goals are set and several behaviors are targeted. Focus on multiple messages and goals may lead to dilution of the intervention intensity or participant fatigue. Further, complex interventions may require a high health literacy level to comprehend and execute. A high proportion of adults (9 out of 10) are reported to lack the skills required to comprehend and apply health related information to improve their well-being [47]. In addition, other dietary approaches require dietary restriction, however, the SG high fiber diet message encourages one to eat ad libitum. Psychologically this may be advantageous. Therefore, a SG high fiber diet intervention with repeated reinforced messages where the direct benefit of making a behavior change can be easily seen may be most effective to aid behavior change and improve health.

Focusing on increasing fiber intake only represents a simple intervention style that may increase adherence. In addition to a simple intervention design, increasing dietary fiber has physiological benefits that aid in prevention of excessive GWG and improvement in maternal health. Fiber exerts protective health benefits that are important during the transient excursion into a metabolic syndrome like state of pregnancy. Increased adiposity and poor diet quality outside of pregnancy play a central role in the deterioration of the metabolic profile contributing to disease development. Fiber effects body weight regulation by increasing the release of satiety hormones leading to reduced hunger [20] and lowering postprandial insulin and glucose responses that promote lipolysis and lipid oxidation over fat storage [48]. Therefore, improving the glycemic profile and decreasing adipose tissue accrual may help prevent metabolic dysfunction during pregnancy (e.g., gestational diabetes, pre-eclampsia, gestational hypertension) that is linked to an acceleration of lifetime risk for vascular and metabolic disease [41]. Therefore, combining a simple intervention style may increase adherence, with further benefit being provided by increasing fiber intake, which has many numerous known health benefits and is protective against weight gain and fat accrual.

One way these effects are accomplished is through the powerful prebiotic effect of fiber to change the gut microbiota [23], gastrointestinal processes (e.g., gastric emptying rate, small intestine and colonic transit time, and intestinal permeability) [24], and the microbial metabolome [25]. Considering the beneficial effect of consuming fiber coupled with low dietary fiber intake in the United States during pregnancy, an intervention targeting increased dietary fiber intake has a large potential for significant impact.

There are limitations to the current study. This was a pilot study and the sample size was limited, however, body weight, body composition, and dietary data showed meaningful differences during the intervention and postpartum. Further, differences found were in the same direction and were similar between the total sample and those classified as compliant. Second, the amount of GWG may not be generalizable to interventions that do not include snacks or a method to increase dietary fiber intake early in pregnancy. Third, while the simplicity of a SG intervention holds promise for clinical implementation, future studies are needed to understand the feasibility and best format for delivery in a clinical setting.

## Conclusions

The SG high fiber intervention resulted in less weight gain and fat accrual during pregnancy and less weight retained at 1 year postpartum. Further, women reported continuing to try and consume a high fiber diet and using multiple skills taught during the intervention. Success of a SG high fiber intervention may be attributed to the focus on one goal coupled with the numerous known benefits of fiber related to weight management. Therefore, a SG high fiber dietary intervention holds promise to reduce weight gain, fat accrual and postpartum weight retention and for clinical implementation, however, further testing in a larger sample must occur.

## Abbreviations

FM: Fat mass
GWG: Gestational weight gain
SG: Single goal
UC: Usual care
TBW: Total body water
BV: Body volume
% fat: Percentage of FM
MG: Multiple
GI: Glycemic index
ROLO: Randomized Control Trial of Low Glycemic Index Diet
